# Randomized, placebo-controlled trial evaluating the safety of one-year administration of green tea catechins

**DOI:** 10.18632/oncotarget.12222

**Published:** 2016-09-23

**Authors:** Nagi B. Kumar, Julio Pow-Sang, Philippe E. Spiess, Jong Park, Raoul Salup, Christopher R. Williams, Howard Parnes, Michael J. Schell

**Affiliations:** ^1^ H. Lee Moffitt Cancer Center & Research Institute, Inc., Cancer Epidemiology, Tampa, FL 33612-9497, USA; ^2^ H. Lee Moffitt Cancer Center & Research Institute, Inc., Genitourinary Oncology, Tampa, FL 33612-9497, USA; ^3^ University of South Florida College of Medicine, Urology, Tampa, FL 33612, USA; ^4^ University of Florida – Jacksonville, UF Health Jacksonville, Urologic Oncology, Jacksonville, FL 32209, USA; ^5^ National Cancer Institute, Division of Cancer Prevention, Bethesda, MD 20892, USA; ^6^ Moffitt Cancer Center & Research Institute, Inc., Biostatistics and Bioinformatics, Tampa, FL 33612-9497, USA

**Keywords:** green tea, catechins, safety, prostate cancer

## Abstract

**Purpose:**

Although preclinical, epidemiological and prior clinical trial data suggest that green tea catechins (GTCs) may reduce prostate cancer (PCa) risk, several preclinical studies and case reports have reported liver toxicities and acute gastrointestinal bleeding. Based on these observations, regulatory bodies have required stringent inclusion criteria with frequent, excessive toxicity monitoring and early stopping rules in clinical trials. These requirements have impeded recruitment and retention of subjects in chemoprevention trials and subsequent progress in agent development efforts.

**Experimental Design:**

We conducted a placebo-controlled, randomized clinical trial of Polyphenon E® (PolyE®), a proprietary mixture of decaffeinated GTCs, containing 400 mg (−)-epigallocatechin-3-gallate (EGCG) per day, in 97 men with high-grade prostatic intraepithelial neoplasia (HGPIN) and/or atypical small acinar proliferation (ASAP). PolyE® containing 200 mg EGCG was administered with food, BID. A secondary study endpoint in this trial was a comparison of the overall one-year treatment related adverse events and grade 3 or higher adverse event on the two study arms. Monthly assessments of toxicity (CTCAE 4.0), concomitant medications and organ function, including hepatic panel, PT/PTT and LDH, were performed.

**Results:**

Daily intake of a standardized, decaffeinated, catechin mixture containing 200 mg EGCG BID taken with food for 1 year accumulated in plasma and was well tolerated and did not produce treatment related adverse effects in men with baseline HGPIN or ASAP.

**Conclusion:**

The current data provides evidence of safety of decaffeinated, catechin mixture containing 200 mg EGCG BID to be further tested for prostate cancer prevention or other indications.

## INTRODUCTION

Green tea, extracted from the leaves of the Camelia sinensis species of the Theaceae family has been extensively consumed as a beverage in Asian countries such as China, Japan, Korea, and India for centuries [1–5]. The catechins present in Green tea include (−)-epigallocatechin-3-gallate (EGCG), (−)-epicatechin (EC), (−)-epigallocatechin (EGC), and (−)-epicatechin-3-gallate (ECG) [32]. Among these green tea catechins (GTC), *in vitro* and animal studies have demonstrated that (−)-epigallocatechin-3-gallate (EGCG) is bioavailable and targets the molecular pathways implicated in prostate carcinogenesis. [2, 4–7]. GTC tested in preclinical [2–4, 7–10] and Phase I/II studies for durations of interventions no longer than 3 months,, have demonstrated bioavailability at doses anywhere between 200-1200 mg EGCG per day [11–17]. Several caveats critical to ensure safety in the design of phase II clinical trials have evolved from these earlier reports. In animal and phase I clinical trials, it has been shown that bioavailbility is increased when GTCs are consumed in a fasting state [18]. However, toxicity, including hepatotoxicity is increased. [19–27]. In these cases, terminating administration of GTC normalized liver function while restarting supplementation with GTC elevated markers of hepatotoxicity [28, 29]. In addition, the National Cancer Institute's Division of Cancer Prevention communicated a case of acute rectal bleeding with EGCG supplements. On the other hand, Pisters et al. [30] reported that the neurological and gastrointestinal adverse events observed with oral green tea extracts administered to adult patients with solid tumors were caffeine-related, and not from catechins. However, Bettuzzi et al. reported no toxicities with green tea catechins in men with HGPIN randomized to receive 300 mgs EGCG per day for one year [10–11]. Although this was the only clinical trial of one-year duration, the study had several limitations. For example, GTC supplement used in the study were not put through rigorous testing for stability of catechin content over the duration of the trial. The study, in addition, did not collect nor report objective data on compliance, adherence or dose-related toxicity, including liver toxicity [10, 11]. Current data fail to provide safety and tolerance information with substantial duration of exposure to GTC, relevant for clinical application for chemoprevention. Despite the lack of evidence, the use of various formulations of GTC as a dietary supplement or as an ingredient in functional foods have increased significantly over the past decade [31], leading to concerns regarding safety with prolonged use of GTC. Based on the toxicities reported in case reports and preclinical data, the Food and Drug Administration (FDA), has imposed stringent criteria for subject selection, periodic and comprehensive monitoring of toxicity and rules for early stopping of study agent for all grades of toxicity, including for grades I-II. This has impeded recruitment and retention of populations at high risk for cancer in well-designed clinical trials using GTC. With the current evidence supporting the evaluation of GTC for cancer chemoprevention in larger cohorts of men diagnosed with precursor lesions (high-grade prostatic intraepithelial neoplasia) or on active surveillance, which may require longer periods of interventions [32], underscores the need for information on safety of prolonged use of GTC in well-designed clinical trials is much needed.

To further evaluate the effectiveness and safety of GTCs for prostate cancer chemoprevention, administered for a duration of 12 months, we conducted a randomized, trial of Polyphenon E® (PolyE), a proprietary mixture of green tea catechins, in 97 men diagnosed with atypical small acinar proliferation (ASAP) or/and HGPIN [32]. Each capsule of PolyE contained 200 mg epigallocatechin gallate, 48.5 mg epigallocatechin, 34.2 mg epicatechin, 20 mg epicatechin gallate, and other tea catechins, 28.8 mg pregelatinized starch, 2.25 mg colloidal silicon dioxide, and 2.25 mg magnesium stearate (Mitsui Norin Co., Ltd., Shizuoka, Japan), in compliance with current good manufacturing practice regulations. The effectiveness of the agent compared to placebo has been reported previously by our team [18, 32]. Secondary outcome measures that were proposed in this trial included a comparison of treatment emergent adverse events (AEs) with 12 month administration of PolyE as measured by the number of participants with AEs possibly or probably related to treatment and grade 3 or higher adverse event on the two study arms [32].

## RESULTS

After screening men for eligibility, ninety seven men (97) were randomized to the trial, with 49 subjects randomized to the treatment arm (PolyE) group and 48 to the placebo group of the trial. Seventy (70) subjects completed the full one year intervention and an additional four reached the six months point with a biopsy at that point. The demographic data regarding the study subjects are displayed in Table 1. The two study arms were matched equally on biomarkers of known risk for prostate cancer. Based on the results, concentrations of plasma EGCG were significantly higher in men in the treatment arm compared to those in the placebo arm of the trial. (Table 2). Change in plasma EGCG concentrations in individual subjects was observed to be greater in the treatment arm (Poly E) at 6 and 12 months compared to placebo. In spite of multi-level verification of compliance by the use of pill counts and diaries, and specific instruction to take the agent 1-2 hours prior to coming in for blood draw to determine catechin levels, individual EGCG concentrations decreased in subjects in the PolyE arm after 6 months. We were unable to detect or other catechins other than EGCG or they were below quantifiable concentrations in subjects in both the treatment and placebo arms of the trial. Nutrient intake of macro and micronutrients in both the treatment and placebo arms remained unchanged from start to end of study [32].

**Table 1:** Demographic characteristics of all study participants randomized to the clinical trial (N= 97)

Variables	Levels	Poly E (N=49)	Placebo (N=48)	P value*
		N (%)	N (%)	
Age (years)	Mean (SD)	62.0 (7.9)	64.1 (7.9)	0.24
Race	Black Or African American	8 (16.3)	12 (25.0)	0.32
	White	41 (83.7)	36 (75.0)	
Ethnicity	Hispanic	6 (12.2)	3 (6.3)	0.40
	Non-Hispanic	42 (85.7)	45 (93.8)	
	Unknown	1 (2.0)	0 (0.0)	
Family History of Prostate Cancer	N	42 (85.7)	45 (93.8)	0.32
	Y	7 (14.3)	3 (6.3)	
Body Mass Index (Weight in Kgs/height in m^2^)	Mean (SD)	29.6 (4.9)	29.8 (4.9)	0.91
PSA (ng/ml)	Mean (SD)	4.5 (1.8)	4.6 (2.1)	0.67
Subjects with baseline HGPIN		32(65.3)	34(70.8)	0.66
No. of cores with baseline HGPIN	Mean (SD)	1.8 (1.4)	2 (1.1)	0.13
Subjects with baseline ASAP		17 (34.7)	14(29.2)	0.66
No. of cores with baseline ASAP	Mean (SD)	1.3 (0.6)	1.5 (0.8)	0.44

Abbreviations: ASAP, atypical small acinar proliferation; HGPIN, high-grade prostatic intraepithelial neoplasia; PSA, prostate-specific antigen; SD, standard deviation.

* P values were computed by Fisher's exact test for categorical variables, Wilcoxon rank-sum test for continuous variables

**Table 2:** Plasma concentrations of EGCG from baseline to post intervention by study arm (N=74)

Time (months)	Placebo				Polyphenon E®				P value*
	N(EGCG>0)	25% Quartile ng/mL	Median ng/mL	75% Quartile ng/mL	N (EGCG>0)	25% Quartile ng/mL	Median ng/mL	75% Quartile ng/mL	
0	0/38	0	0	0	0/36	0	0	0	1
6	2/37	0	0	0	22/35	0	6.6	21.8	<.0001
12	1/31	0	0	0	13/28	0	0	11.5	0.0002

* P value comparing all individual patients' plasma EGCG concentrations at each time point between placebo vs. poly E was calculated from Wilcoxon rank-sum test, 2-sided.

We present in Table 3 toxicity summary by maximum grade (definite, probable, possible, unlikely, and unrelated) and the toxicities by final attribution are presented in Tables 3–4 respectively. Compared to the placebo group, more possible and probable events related to study agent was observed in the men treated with the study agent, Among these events observed, only one (1) event was categorized as a Grade III AE (nausea) that was possibly related to PolyE. All other events were grade I-II metabolic abnormalities such as changes in LFTs observed primarily from monthly laboratory testing (CMP and CBC, LFTs) and not events reported by subjects. Similarly, relatively more events of changes in PT/PTT in the Poly E arm compared to the placebo., Additionally, we did not observe any differences between groups related to serum PT/PTT that were indicative of excessive bleeding or clotting disorders. Subjects in both arms reported musculosketal pain. However, these reports did not differ significantly between the two arms of the study with some of these events present in those using statins (Placebo: 3/5 and Poly E 9/13 events). A greater number of events of pain were reported in the treatment arm compared to placebo, although these symptoms were determined unrelated to study agent. [Placebo: 9 (19%) and Poly E: 17 (35%) (P = 0.08)].

**Table 3:** Summary of Toxicities by Final Attribution and Treatment Arm - All Patients (N=97)

Final Attribution		Treatment Arms	
N (%)	Placebo	Polyphenon E®	Total
**Definite**	0 (0)	0 (0)	0 (0)
**Possible**	3(1.78)	7(3.30)	10(2.62)
**Probable**	1(0.59)	5(2.36)	6(1.57)
**Unlikely**	9(5.33)	7(3.30)	16(4.20)
**Unrelated**	156(92.31)	193(91.04)	349(91.60)
**Total**	169(44.36)	212(55.64)	381(100.0)

**Table 4:** Comparison of all Adverse events by Group and Toxicity Symptom category: (n=97)

Toxicity Category	Placebo # Subjects (%)	Poly E # Subjects (%)	P value*
Allergy/Immunology	4(8)	3(6)	0.77
Auditory/Ear	1(2)	0(0)	**
Blood/Bone Marrow	16(33)	15(31)	0.84
Cardiac Arrhythmia	2(4)	1(2)	**
Cardiac General	1(2)	1(2)	**
**Coagulation**	**5(10)**	**11(22)**	**0.12**
Constitutional Symptoms	9(19)	11(22)	0.71
Death	0(0)	1(2)	**
Dermatology/Skin	3(6)	7(14)	0.24
Endocrine	0(0)	2(4)	**
**Gastrointestinal**	**12(25)**	**17(35)**	**0.31**
**Hepatobiliary/Pancreas**	**1(2)**	**1(2)**	**
Infection	2(4)	5(10)	0.28
Lymphatics	0(0)	1(2)	**
Metabolic/Laboratory	37(77)	34(69)	0.49
**Musculoskeletal/Soft Tissue**	**3(6)**	**9(18)**	**0.07**
Neurology	3(6)	3(6)	**
Ocular/Visual	1(2)	3(6)	**
**Pain**	**9(19)**	**17(35)**	**0.08**
Pulmonary/Upper Respiratory	7(15)	7(14)	1
Renal/Genitourinary	11(23)	11(22)	1
Other Symptoms	1(2)	2(4)	**

* **Barnard test for toxicity category**

** **p-values are not provided if the total number of toxicities was 3 or fewer**.

During the trial period and as directed by Food and Drug Administration, 11 subjects from the treatment arm and 7 in the placebo arm, met off-study criteria due to AEs. (Data not shown). On the other hand, the numbers of subjects with specifically, grade I-II liver toxicity and the off-study criteria set forth by the FDA were not statistically significantly different between the two groups [32]. The single grade IV toxicity was a reported suicide, unrelated to study agent.

We did not observe any change in Lower Urinary Tract Symptoms and Quality of Life scores between the two arms of the study from baseline to end of study.

## DISCUSSION

Our study is the first randomized clinical trial of the safety of a GTC containing 400 mgs EGCG administered in divided doses and non-fasted condition for duration of 1 year. One year administration of 400 mgs EGCG administered in a fed state and in divided doses of 200 mgs EGCG twice a day did not produce liver or other toxicities in men at elevated risk for prostate cancer. With the exception of a single grade III nausea reported that may possibly be related to PolyE, no other dose limiting toxicities were observed at this dose and mode of administration.

In preclinical chronic toxicity studies performed in dogs administered during a fasting state have revealed some distinctive limiting toxicities, including gastrointestinal, hepatic and renal toxicities [21]. The GTC mixture used in these studies were formulated primarily with EGCG [≥55%] in addition to 10% each of epigallocatechin, epicatechin, and epigallocatechin gallate [21]. Using the same dose levels, when these studies were conducted in non-fasted dogs, toxicities were unremarkable, suggesting that the fasting condition may have contributed to the increased vulnerability of the target organs to toxicity with GTC. In a preclinical study [17] targeting TRAMP mice with several doses of a standardized GTC-PolyE, we failed to observe liver or other toxicities. Similarly, in animal studies with EGCG, Isbrucker et al. [37] did not observe toxicities, including liver toxicities. In the past few years, several case reports of liver toxicity including liver failure have been reported with oral green tea extracts when used for weight loss or for other indications [20–25]. The dose of GTC used in these case reports ranged from 375 mgs [22–24] for 4 months to 1.8 grams GTC per day [25], predominantly used as weight loss supplements along with other ingredients. However, the liver toxicities reported have included non-standardized formulations with varying doses, compositions and duration of use of GTC. Additionally, the intake was predominantly self- reported cases with poor documentation of other potential contaminants as well as concomitant medications that may have been consumed with the green tea. The results of our trial, however, failed to demonstrate differences in liver toxicity between the treatment and placebo arms when GTCs were administered at a dose of 200 mgs EGCG BID in a non-fasting state. Based on a case report of rectal bleeding with high doses of GTC, communicated by NCI-DCP, the FDA required addition of a criterion to exclude men with history of medical conditions that may predispose them to gastrointestinal bleeding (acute or chronic gastritis or colitis, or acute diverticulitis or hemorrhoids). However, based on our findings, we did not observe significant differences between toxicities due to bleeding or elevated serum PT/PTT between the two arms of the study. With subjects at risk of gastrointestinal bleeding disorders already excluded from our trial, we are unable to ascertain if the absence of these toxicities were related to this exclusion or if this was simply a symptom that we did not observe. However, other phase I-II clinical trials [11, 13, 18, 35, 38, 39] did not specifically examine these changes and adverse events related to gastrointestinal bleeding with GTC have not been reported. Study subjects in both the treatment and placebo arms reported symptoms of pain including musculoskeletal pain, with several more events reported by men in the treatment arm. These grade I-II events were all in men on statins. Notably, musculoskeletal conditions, arthropathies, injuries, and pain are relatively more common among statin users than among nonusers [40–43]. One of the most common symptoms observed in over 25% of statin users is myopathy characterized by symptoms of cramping, weakness and muscle fatigue [40–43]. Musculoskeletal pain, particularly in the lower extremities, among individuals without arthritis has been reported [40]. Although the mechanisms implicated in statin-induced myopathy have not been elucidated, research suggests that apoptosis of myofibers [42] may contribute to musculoskeletal conditions related to statin use [42]. Although the sample size in this clinical trial was too small to examine and characterize other contributing factors such as physical activity, arthritis or other contributory conditions, future studies should continue to monitor these symptoms. LUTS represent a common cluster of symptoms including of storage, voiding, and post-micturition, with reported debilitating effect on quality of life [32, 33, 44, 45]. LUTS is reported more commonly in men over age 60 and those diagnosed with benign prostatic hyperplasia [11, 12, 32, 33, 44, 45]. Subjects in our clinical trial study were generally asymptomatic at baseline and post intervention in both the treatment arm receiving PolyE® and placebo.

Although findings of chemoprevention interventions with 5-alpha-reductase inhibitors, finasteride and dutasteride have previously reported reduced risk of PCa, their use was also associated with increased detection of high-grade disease, severely limiting their clinical adoption as prostate cancer prevention agents [47]. Based on these observations, we observed rates of PCa at one year, serum PSA as well as Gleason scores of men who progressed to PCa within the year in this study population. Rates of PCa at 1 year as well as Gleason scores in men treated with Poly E were lower compared to the placebo arm. Although the sample sizes were small, these observations indicate that PolyE may not be associated with increased risk of detection of high-grade disease. In men with HGPIN without ASAP at baseline, we did not observe any progression to ASAP (0/26) in the Poly E arm compared to (5/25) in the placebo arm who were subsequently diagnosed with ASAP [32].

In summary, our study provides strong evidence that a daily intake of a standardized, decaffeinated catechin mixture containing 400 mg EGCG per day for 1 year administered with food (non-fasting), in two divided doses, accumulated in plasma, was well tolerated and did not produce treatment related adverse effects in men with ASAP or HGPIN. Additionally, GTC at this dose and duration of intervention was not associated with increased risk of detection of high-grade disease in group of men at end of study (EOS).

## MATERIALS AND METHODS

### Selection and description of participants

The study and the consent procedures were approved by the institutional review boards of all participating institutions. A consort diagram depicting the number of subjects screened, enrolled, randomized and completed intervention as well as detailed materials and methods for this study have been previously reported [32]. Briefly, men between ages 30-80 with a biopsy-proven diagnosis of HGPIN and/or ASAP less than 3 months before randomization, with no history of cancer, hepatic or renal disease, restricted from taking steroid or other supplements, or more than 6-12 cups of green tea a day, were eligible. All prostate biopsies were reviewed by a central pathology laboratory and all pathologists were unaware of the treatment-group assignment. Discordant interpretations were arbitrated by a referee pathologist (senior pathologist at Moffitt Cancer Center), and concordance was achieved in all cases. Participants were enrolled at the Moffitt Cancer Center, James A. Haley VA Hospital, Tampa and University of Florida, Jacksonville, Florida from September 2008 to March 2013.

### Technical information

After eligibility was confirmed and consent obtained, participants were assigned to the intervention or placebo arm (1:1 randomization) using the SRAR system, a web-delivered subject registration application, stratified by diagnosis (HGPIN or ASAP). At randomization, baseline assessments of lower urinary tract symptoms (LUTS) using the LUTS Symptoms Scale [33], quality of life (QOL), using the Rand Short-form (SF)-36 [34], prostate specific antigen (PSA) and plasma catechin levels were obtained. Total serum PSA was determined using radioimmunoassay methods for determining total PSA by Quest Diagnostics. (Tampa, Florida). Blood samples, urine and tissue from diagnostic biopsy were collected for baseline measurements and banked for future studies.

Although increased oral bioavailability occurs when GTCs are consumed in a fasting state [18], increased toxicity has been reported when EGCG is taken in a fasting state than when taken in a fed state. Studies on single doses in fasting and fed conditions using 400, 800 and 1200 mgs EGCG have reported higher plasma EGCG concentrations in fasting conditions relative to fed conditions [19, 21]. Similarly, the bioavailability and tolerance to a multiple dosing schedule compared to a single daily dose of EGCG has been reported [35]. Based on these studies, participants were instructed to take the agent in divided doses of one capsule (200 mg EGCG) twice a day on a full stomach, one capsule with breakfast and one with dinner, to provide a continuous dose of EGCG while reducing any gastrointestinal symptoms and to minimize toxicity. With reports of liver toxicity in clinical and preclinical trials [22–27], the FDA also required liver function tests performed at baseline and repeated every four weeks while subjects were on treatment. Following any elevation in ALT, the FDA required that study drug be withheld (grade 1) or discontinued (grade 2), and liver function monitored until recovery to normal. With reports of rectal bleeding reported in case study of a woman who took mega-doses of EGCG for weight loss, criteria for exclusion included those subjects with any chronic rectal bleeding. Serum PT/PTT were also monitored monthly for excessive bleeding or bleeding disorders based on a case report of rectal bleeding (personal communication from DCP) with high doses of GTC. Criteria for exclusion included men with history of medical conditions that may predispose the subject to gastrointestinal bleeding (acute or chronic gastritis or colitis, or acute diverticulitis or hemorrhoids).

LUTS [31], QOL [34], plasma catechin levels, PSA and nutritional intake data were evaluated at baseline, 3 and 6 months, and at end-of-study (EOS). Monthly assessments of toxicity (CTCAE 4.0), concomitant medications and organ function, including hepatic panel, PT/PTT and LDH, were performed. Repeat biopsies were performed at six months for: (a) PSA velocity >0.75ng/ml, or; (b) documentation of a prostate nodule on digital rectal examination. All participants who did not have PCa detected on an interim biopsy underwent EOS biopsy at 1 year. Any toxicities (adverse events) occurring during the study were reviewed by the treating physician and managed according to standard medical practice. The intervention was terminated if a participant developed PCa or a serious adverse event. All subjects were contacted 7±3 days following the 1-year intervention to assess toxicity and concomitant medications. Toxicities were monitored continuously through the trial by the PI and study physician at each site. The study was monitored following a monitoring plan developed by the Protocol Review and Monitoring System at the Moffitt Cancer Center and an External Data and Safety Monitoring Board (EDSMB).

Compliance with study agent intake was measured during monthly visits via pill counts and self-reported daily study-agent intake logs. Adherence was assessed by measuring plasma catechin levels at baseline, 6 months and EOS. A validated liquid chromatography triple quadrupole mass spectrometry (LC/MS/MS) method (Thermo Scientific, San Jose, CA) was used to determine plasma catechin levels. We were able to successfully quantitate the four catechins (EGCG, EGC, ECG and EC) using methods previously described [13, 18, 35].

Results of the primary endpoint comparing the cumulative number of PCa diagnoses at 1-year on the two study arms have been published. Secondary endpoints included comparisons of overall: (a) treatment-related adverse events; (b) AEs definitely, possibly or probably related to treatment; and (c) AEs grade 3 or higher per treatment arm from baseline to 6 and 12 months. (Clinical Trials.gov NCT00596011).

### Statistical analysis

Baseline participant characteristics were compared between the two groups using Fisher exact tests for categorical variables and Wilcoxon rank-sum test for continuous variables. Trend for adverse events by group, grade and causality were compared using the Jonckheere-Terpstra test and toxicity symptoms using the Barnard unconditional test [36] Plasma EGCG levels, nutritional intake, LUTS and QOL were compared by study arm from baseline to end of intervention using 2-sided Wilcoxon rank-sum test. The overall rate of PCa diagnoses among men with HGPIN or ASAP on baseline biopsy in the two treatment groups was compared using the log-rank test, with event times at either 6 or 12 months. A pre-specified secondary endpoint comparing the cumulative 1-year rate of PCa plus ASAP among men with HGPIN without ASAP at baseline was performed using the Barnard unconditional test, as no cases of PCa were detected before the 12 month EOS biopsy in this group. An intention-to-treat analysis was used for the primary efficacy endpoint. We estimated the overall treatment effect on serum PSA between the two arms using the GEE model which accounts for all 74 patients with PSA values at 6 and 12 months. To assess the effect of treatment on PCa grade in subjects who developed PCa while on study, we compared Gleason categories using a Fisher exact test at α = .05 for the 2×4 contingency table.
